# Physical Non-Contact Communication between Microscopic Aquatic Species: Novel Experimental Evidences for an Interspecies Information Exchange

**DOI:** 10.1155/2016/7406356

**Published:** 2016-03-02

**Authors:** Daniel Fels

**Affiliations:** University of Basel, Botanical Institute, Hebelstrasse 1, 4056 Basel, Switzerland

## Abstract

Previous experiments on physical non-contact communication within same species gave rise to test for this type of communication also across the species border, which was the aim of the present study. It was found that autotrophic unicellular organisms (*Euglena viridis*), separated by cuvettes, affected the proliferation rate of heterotrophic unicellular organisms (*Paramecium caudatum*). Further, the heterotrophic unicellular organism affected also the proliferation rate of a multicellular heterotrophic organism (*Rotatoria *sp.) and vice versa. In the case when populations (of* Euglena viridis *and* Paramecium caudatum*) were shielded against electromagnetic fields in the optical spectrum from each other, no effects were measured. The results may support the notion that the organisation of ecosystems relies also on the exchange of electromagnetic fields from their constituting biosystems.

## 1. Introduction

Functional coordination between cells (or unicellular organisms) is mediated by complex processes of information exchange. The carriers of this communication are generally chemical (i.e., neurotransmitters and autoinducer molecules) or physical (e.g., electrical signals) in nature. Despite the well-known chemical information exchange in a* non-contact* mode between cells, there are experimental evidences starting almost hundred years ago that there is also a physical (i.e., nonchemical) non-contact communication taking place between cells [1]. Pioneering researchers in this regard were the Russian scientists A. Gurvitsch and V. P. Kaznacheev who concluded that there could be an optical (i.e., electromagnetic) cell-to-cell communication (for reviews about this topic see [2–4]).

In order to test these claims with novel modern experiments (e.g., [5, 6]), the present author conducted experiments with* Paramecium caudatum* populations and could show that indeed intercellular non-contact and nonchemical interaction between the* Paramecium caudatum* populations could be detected when separated with a glass barrier (or either quartz and normal glass) [7, 8]. The characteristics of effects were dependent on the type of glass used and numbers of cells involved.

These results motivated the author to hypothesize that a physical non-contact communication (possible via electromagnetic radiation) may have an important role in cell-interactions also within ecological systems (see also [9]). In particular, it was hypothesized that there is a non-contact electromagnetic interaction that is taking place not only between the same species but also between* different* ones. Such effects were indeed already reported, for example, from bacteria on egg cells of sea urchins or from onion root cells on frog egg cells (e.g., [10, 11]) but were not repeated with modern experimental setups yet. In order to continue this research, the aim of the present study was to investigate if there is such a non-contact and nonchemical interaction between three* different* aquatic unicellular eukaryotic species (*Euglena viridis*,* Paramecium caudatum*, and* Rotatoria *sp.).

## 2. Materials and Methods

The experiments were performed using cuvettes of two sizes, where the smaller (15 mm × 15 mm × 45 mm) could be placed into the bigger (23 mm × 23 mm × 40 mm). This separates populations chemically but not physically, in particular electromagnetically [7, 12]. The cuvettes consisted in either quartz or glass, which latter could serve as a filter for UV-B and UV-C and may therefore give different results as compared to separation with quartz [7].

The pairs of cuvettes (i.e., small cuvettes placed in the larger ones) containing controls (in one case with graphite shielding) and treatment were randomly placed in a grid where the pairs were at close vicinity but separated from each other by light-barriers.

All populations, that is, inducer and tester (receiver) populations, were kept in a light-safe box during mutual exposure. Exposure lasted 48 hrs with the light-safe box standing in an incubator at 24°C (but 22°C in experiment  1b). At the end of an experiment the number of individuals in tester populations was counted and used for data analysis. Sizes of tester populations were counted with the help of a binocular microscope.

The organisms were* Paramecium caudatum*, a unicellular ciliate of about 250 *μ*m length,* Euglena viridis*, a unicellular flagellate of about 60 *μ*m length, and* Rotatoria *sp., a multicellular organism of about 500 *μ*m length. All of them inhabit ponds or lakes being potential conspecifics.* Paramecia *belong to the author's own cultures and were fed with bacteria (for more information please refer to [7]). The photosynthetic* Euglena* came from a lab stock and was kept in standard mineral water.* Rotatoria *were originally taken from a contaminated protozoan culture in the lab. They were fed like* Paramecia *with bacteria.

### 2.1. Experiment  1

#### 2.1.1. Experiment  1a: Interspecies Communication between* Paramecia* and* Euglena*

This experiment tested for an effect of* Euglena viridis* on* Paramecia caudatum*. The big cuvettes (BC) contained within 1 mL water about 100,000* Euglena* cells. In the small cuvette (SC) there were at the onset of the experiment 100* Paramecia*. Controls had just 1 mL of mineral water in the BC.* Paramecia* were in their own medium (see [7]). The cuvette pairs consisted either in glass or in quartz. An experimental block consisted in five replicates of each treatment group and was repeated three times. The assessment was the cell division rate, that is, the population size of* Paramecia *at the end of the exposure time.

#### 2.1.2. Experiment  1b: Interspecies Communication between* Paramecia* and* Euglena *with Graphite Shielding

Shielding is a commonly used method when looking for electromagnetic effects between organisms or cells (e.g., [5, 13, 14]). If the signal coming from* Euglena *and affecting* Paramecium caudatum* is electromagnetic, then a thin layer of colloidal graphite around the inner cuvette should prevent electromagnetic signals [15] coming from the outer inducer population that could induce an effect on the inner tester population. Using purest colloidal graphite in solution (CRAMOLIN® GRAPHIT) a graphite-layer was twice sprayed onto the bottom and up to a height of 15 mm on the outer side of the small cuvettes (the volume of 1 mL reaches in a cuvette pair only 4 mm in the outer and 6 mm in the inner cuvette).

Only quartz cuvettes were used in this variation of Experiment  1a and the density of* Euglena viridis *was 250,000/mL. The design was otherwise as described above with (despite the* normal* control) the additional control where the inner population of* Paramecium *was shielded with graphite from* Euglena *in the outer cuvette. The experimental block consisted in five replicates of each of the three treatment groups and was repeated four times.

### 2.2. Experiment  2: Interspecies Communication between* Paramecia* and* Rotatoria*

In the BC there were 100* Rotatoria *in 1 mL medium. In the SC there were 100* Paramecia *in 1 mL medium. There were two controls, one for* Rotatoria* (with medium only in the SC) and one for* Paramecia* (with medium only in the BC). Separation occurred with both glass and quartz cuvettes. Controls and treatment cuvette pairs were randomly placed in a grid where the pairs were at close vicinity but separated from each other by light-barriers. The assessment was the population size of* Paramecia *and that of* Rotatoria *at the end of the exposure time.

### 2.3. Statistics

Log-transformed data of population sizes of* Rotatoria* and* Paramecia* were used to perform an analysis of variance (ANOVA).

## 3. Results

### 3.1. Experiment  1

#### 3.1.1. Experiment  1a: Interspecies Communication between* Paramecia* and* Euglena*

**Figure 1:**
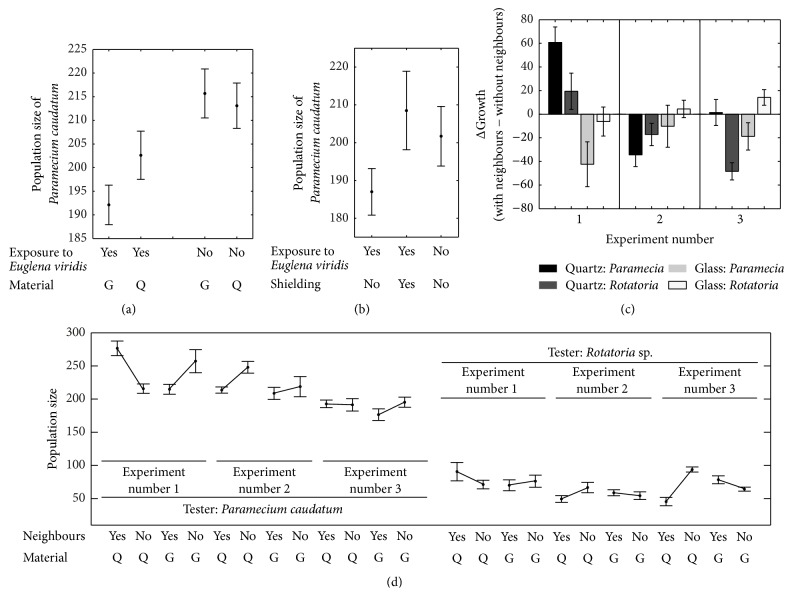
(a–d) represent graphically the results from experiments  1a and 1b and 2. (a) Glass- and quartz-separated populations of* Euglena viridis* affecting populations of* Paramecium caudatum*. (b) As in (a) but with graphite shielding and quartz-separation only. (c) refers to experiment  2 and displays the effect of* Rotatoria *sp. on* Paramecium caudatum *andvice versa. The *x*-axis shows the three repetitions of experimental blocks and the *y*-axis values of growth with inducer species as neighbour minus growth of controls. (d) refers to the same experiment as in (c) but shows the actual growth values of each treatment group for all three repetitions of experimental blocks.

The presence of* Euglena *cells had a significant effect on growth performance in* Paramecia*: they displayed a retarded cell division rate when neighboured by* Euglena* (Tables 1 and 2 and Figure 1(a)). Otherwise there were no effects coming from repeating the experiment (statistics not shown) or from using different separating material (Table 2). After 48 hrs of exposure the BC with* Euglena *showed green fogy stripes in the medium, most probably chloroplasts that are known to be expulsed under conditions of darkness.

#### 3.1.2. Experiment  1b: Interspecies Communication between* Paramecia* and* Euglena *with Graphite Shielding

The presence of* Euglena *cells had the same growth retarding effect as in Experiment  1 (Tables 3 and 4 and Figure 1(b)). There were also effects coming from repeating the experiment (Table 4). The shielded Paramecium population grew as well as the control population that had no neighbouring inducer population (a contrast analysis showed no difference in growth performance between the two controls: *p* > 0.241). There was no indication for expulsed chloroplasts.

### 3.2. Experiment  2: Interspecies Communication between* Paramecia* and* Rotatoria*

As there were no material effects but material-neighbour interactions (statistics not shown) the data (Table 5) and their analysis was performed with separating them by material (Table 6). The multicellular* Rotatoria *sp.and the unicellular* Paramecium caudatum *have significant effects on each other. In one experimental replication and when separated by quartz the effect was enhancing; otherwise both organisms rather had a reducing effect on the others' proliferation rate (Table 6 and Figures 1(c) and 1(d)).

## 4. Discussion

The results deliver three observations of non-contact and non-chemically induced effects across the species border of two unicellular and one multicellular aquatic organism. The effects, some of which being highly significant, consisted mainly in decreasing the other species' proliferation rate.

It is assumed that the effects were physically transmitted since populations were separated with barriers that disable solvable chemicals from trespassing. Further, volatile substances would not make evolutionary sense to an aquatic organism, and, if existing, any volatile substance would need to solve back into* the right cuvette*. This latter is extremely improbable as the cuvette pairs were very close to each other and, furthermore, treatment groups and controls were randomly placed in a side-by-side manner. In addition, those treatment groups where the inner populations (of* Paramecium*) were shielded with graphite from the outer population (of* Euglena*) showed no effect on the tester population giving two important indications. First, if these populations would really use volatiles that would solve back within the same pair of cuvettes, then this should also happen for the treatment group with a graphite shield, which nonetheless is not supported by the data. Second and this was the major reason for the shielding control, as graphite shields electromagnetic waves and no effect from the inducer population was found, this delivers indirect evidence for an electromagnetic signal inducing observed effects.

As the experiments had taken place in total darkness and such darkness does not exist under natural conditions, the effects between the organisms may not have resulted from natural selection. They may, nonetheless, reflect the use of a universal physical code of life.

It is concluded that between the three different species there has been a physical communication. As discussed elsewhere and as graphite shielding gave indirect evidence, it is assumed that this physical communication is of electromagnetic nature [1] implying that theories on electromagnetic (despite chemical) organisation of whole ecosystems may put us on the right track.

## Figures and Tables

**Table 1:** Listed are the mean values and standard errors (*n* = 15) of the absolute population size of *Paramecium caudatum *at the end of the exposure to *Euglena viridis *or its absence (yes/no). The separation between the species was conducted with cuvettes made of glass (G) or quartz (Q).

Exposure to *Euglena*	Material	Mean ± standard error
Yes	G	192.1 ± 4.2
Yes	Q	202.6 ± 5.1
No	G	215.7 ± 5.2
No	Q	213.1 ± 4.8

**Table 2:** This table refers to the exposure Experiment  1a with *Euglena viridis* on *Paramecium caudatum* and displays the results of an analysis of variance based on final population size. The ANOVA table displays the sum of squares (SS), degrees of freedom (df), *F*-ratio, and the probability of error (prob > *F*).

Source	df	SS	*F*-ratio	Prob > *F*
Material (G/Q)	1	0.006	0.731	0.3961
*Euglena *(y/n)	1	0.104	12.566	0.0008^*∗∗∗*^
Material × *Euglena*	1	0.015	1.843	0.1800

^*∗∗∗*^ *P* < 0.001.

**Table 3:** This table refers to Experiment  1b and lists mean values and standard errors (*n* = 20) of the absolute population size of *Paramecium caudatum *at the end of the exposure to *Euglena viridis *or its absence (yes/no). The separation between the species was conducted with cuvettes made of quartz and had two types of controls (*normal* and shielded; see text).

Exposure to *Euglena*	Shielding	Mean ± standard error
Yes	No	187.0 ± 6.2
Yes	Yes	208.5 ± 10.4
No	No	201.7 ± 7.9

**Table 4:** This table refers to the exposure Experiment  1b (graphite shielding; see text) with *Euglena viridis* on *Paramecium caudatum* and displays the results of an analysis of variance based on final population size. The ANOVA table displays the sum of squares (SS), degrees of freedom (df), the *F*-ratio, and the probability of error (prob > *F*).

Source	df	SS	*F*-ratio	Prob > *F*
Repeating the experiment (RE)	1	1.256	38.727	<0.0001^*∗∗∗∗*^
*Euglena *(y/n)	1	0.127	5.862	0.0053^*∗∗*^
RE × *Euglena*	1	0.063	0.966	0.459

^*∗∗*^ *P* < 0.01, ^*∗∗∗∗*^ *P* < 0.0001.

**Table 5:** This table shows the mean values and standard errors (SE) for each treatment group, that is, for *Paramecium caudatum *with or without *Rotatoria *sp. as neighbours as well as for *Rotatoria *sp. with or without *Paramecium caudatum *as neighbours.

Tester	Experiment	Material	Neighbours	
			Yes	No
			Mean ± SE	Mean ± SE
*Paramecium caudatum*	1	Quartz	276.6 ± 11.0	215.8 ± 7.1
		Glass	214.8 ± 7.5	257.2 ± 17.4
	2	Quartz	213.6 ± 4.7	248.0 ± 8.8
		Glass	208.6 ± 9.2	218.8 ± 15.2
	3	Quartz	192.8 ± 5.7	191.4 ± 9.4
		Glass	176.6 ± 8.7	195.4 ± 7.6

*Rotatoria *sp.	1	Quartz	90.6 ± 13.9	71.2 ± 6.5
		Glass	70.0 ± 8.2	76.2 ± 9.1
	2	Quartz	49.4 ± 5.1	66.6 ± 7.9
		Glass	58.8 ± 4.5	54.4 ± 5.8
	3	Quartz	45.4 ± 6.2	93.8 ± 3.8
		Glass	78.4 ± 5.9	64.2 ± 3.0

**Table 6:** This table shows the results from an analysis of variance coming from the experiment on mutual effects of *Paramecium caudatum *and* Rotatoria *sp. on each other. The ANOVA table displays the sum of squares (SS), degrees of freedom (df), the *F*-ratio, and the probability of error (prob > *F*) based on final population size. Effects came from repeating the experiment (exp), the inducer species (ind), and interactions between repeating the experiment and inducer species (exp × ind).

Tester	Material	Source	df	SS	*F*-ratio	prob > *F*
*Paramecium caudatum*	Quartz	exp	2	0.302	22.677	<0.0001^*∗∗∗∗*^
		ind	1	0.008	1.134	0.2975
		exp × ind	2	0.183	13.728	0.0001^*∗∗∗*^
	Glass	exp	2	0.276	10.163	0.0006^*∗∗∗*^
		ind	1	0.085	6.237	0.0198^*∗*^
		exp × ind	2	0.022	0.810	0.4565

*Rotatoria *sp.	Quartz	exp	2	0.559	4.347	0.0245^*∗*^
		ind	1	0.585	9.090	0.0060^*∗∗*^
		exp × ind	2	1.166	9.062	0.0012
	Glass	exp	2	0.393	4.548	0.0211^*∗*^
		ind	1	0.034	0.782	0.3852
		exp × ind	2	0.096	1.111	0.3456

^*∗*^ *P* < 0.05, ^*∗∗*^ *P* < 0.01, ^*∗∗∗*^ *P* < 0.001, ^*∗∗∗∗*^ *P* < 0.0001.
